# Does inter-vertebral range of motion increase after spinal manipulation? A prospective cohort study

**DOI:** 10.1186/s12998-014-0024-9

**Published:** 2014-07-01

**Authors:** Jonathan Branney, Alan C Breen

**Affiliations:** 1 Institute of Musculoskeletal Research & Clinical Implementation, Anglo-European College of Chiropractic, 13-15 Parkwood Road, Bournemouth BH5 2DF, UK; 2 The School of Health & Social Care, Bournemouth University, Royal London House, Christchurch Road, Bournemouth BH1 3LT, UK

**Keywords:** Neck pain, Manipulation, Spine kinematics, Fluoroscopy, Patient-reported outcomes

## Abstract

**Background:**

Spinal manipulation for nonspecific neck pain is thought to work in part by improving inter-vertebral range of motion (IV-RoM), but it is difficult to measure this or determine whether it is related to clinical outcomes.

**Objectives:**

This study undertook to determine whether cervical spine flexion and extension IV-RoM increases after a course of spinal manipulation, to explore relationships between any IV-RoM increases and clinical outcomes and to compare palpation with objective measurement in the detection of hypo-mobile segments.

**Method:**

Thirty patients with nonspecific neck pain and 30 healthy controls matched for age and gender received quantitative fluoroscopy (QF) screenings to measure flexion and extension IV-RoM (C1-C6) at baseline and 4-week follow-up between September 2012-13. Patients received up to 12 neck manipulations and completed NRS, NDI and Euroqol 5D-5L at baseline, plus PGIC and satisfaction questionnaires at follow-up. IV-RoM accuracy, repeatability and hypo-mobility cut-offs were determined. Minimal detectable changes (MDC) over 4 weeks were calculated from controls. Patients and control IV-RoMs were compared at baseline as well as changes in patients over 4 weeks. Correlations between outcomes and the number of manipulations received and the agreement (Kappa) between palpated and QF-detected of hypo-mobile segments were calculated.

**Results:**

QF had high accuracy (worst RMS error 0.5o) and repeatability (highest SEM 1.1o, lowest ICC 0.90) for IV-RoM measurement. Hypo-mobility cut offs ranged from 0.8o to 3.5o. No outcome was significantly correlated with increased IV-RoM above MDC and there was no significant difference between the number of hypo-mobile segments in patients and controls at baseline or significant increases in IV-RoMs in patients. However, there was a modest and significant correlation between the number of manipulations received and the number of levels and directions whose IV-RoM increased beyond MDC (Rho=0.39, p=0.043). There was also no agreement between palpation and QF in identifying hypo-mobile segments (Kappa 0.04-0.06).

**Conclusions:**

This study found no differences in cervical sagittal IV-RoM between patients with non-specific neck pain and matched controls. There was a modest dose-response relationship between the number of manipulations given and number of levels increasing IV-RoM - providing evidence that neck manipulation has a mechanical effect at segmental levels. However, patient-reported outcomes were not related to this.

## Background

Neck pain is a common condition which most people experience at some point in their lives, with self-reported incidence rates ranging from 15.5 to 213 per 1000 person years [[1]] and 12-month prevalence rates around 30-50%. The condition can also be a significant cause of work absence [[2],[3]], decreased productivity [[4]] and increased healthcare costs [[5]].

Spinal manipulation, mobilisation, exercise, analgesics, acupuncture and low level laser therapy have all been shown to provide at least some degree of short-term relief of neck pain in the absence of trauma [[6]]. Spinal manipulation or mobilisation, particularly combined with exercise, appears to confer marginal benefit over other interventions [[7],[8]]. However, trials investigating manipulation for neck pain have shown conflicting results [[9],[10]] and while some patients respond well, others derive little benefit. One step towards understanding this would be to further our knowledge of the mechanism of action of manipulation as an intervention.

There is evidence to suggest that manipulation can decrease pain and improve function and that this is probably associated with increased motion. Nansel et al. showed that a single manipulation could reduce asymmetry of passive regional cervical spine motion [[11]], while Cassidy et al. and Martinez-Segura et al. independently found that manipulation was associated with both reduced neck pain and increased regional range of neck motion immediately after treatment [[12],[13]]. However, regional neck range of motion is also influenced by pain, disability and fear of movement [[14]], leaving the role of manipulation ambiguous. Furthermore, spinal manipulation targets specific levels, often to improve inter-vertebral range of motion (IV-RoM) [[15]], but it is not known if this actually happens, or if reduced IV-RoM is even detectable by clinical examination (palpation). To begin to explain the therapeutic effects of neck manipulation therefore requires an accurate and reproducible means of measuring maximum IV-RoM wherever it occurs in the neck bending sequence, and relating it to symptomatic changes following a course of manipulative treatment. It is also necessary to know whether reduced motion at inter-vertebral levels as detected by palpation as the basis for a manipulation can be verified.

Cineradiography and videofluoroscopy have allowed the visualisation of complete inter-vertebral motion sequences and have been available for many years [[16],[17]], but analysis of the motion has been largely qualitative. However, during the last decade considerable progress has been made with detailed measurement of inter-vertebral motion using quantitative fluoroscopy (QF) [[18]]. It is now possible to track fluoroscopic images of lumbar vertebrae continuously throughout lumbar motion in living subjects [[19]] allowing the measurement of true IV-RoM, regardless of where it occurs during bending. QF has been found to have good reproducibility for this [[20]], but has not yet been validated or extensively used in the cervical spine. This study aimed to explore the effects of neck manipulation on IV-RoM in the cervical spine as measured by QF.

The study had four main objectives:

1. To determine the accuracy, measurement precision and minimal detectable change (MDC) in IV-RoM over a period of treatment

2. To determine whether cervical spine flexion and extension IV-RoM increases after a course of spinal manipulation for non-specific neck pain and if so, the dose–response associated with such change

3. To determine if there is any correlation between IV-RoM changes and patient reported outcomes

4. To compare the frequency of finding inter-vertebral motion hypomobility by palpation and QF measurement.

## Methods

### Study design

This was a prospective study of a cohort of patients with nonspecific neck pain receiving spinal manipulation and a matched cohort of healthy volunteers as a reference group which took place between August 2011 and April 2013. The primary outcome measure was IV-RoM change after a period of manipulative treatment to the neck.

The study anticipated a number of sources of bias, such as the influence of osteoarthritic changes with age [[21]], possible gender effects on neck pain disability [[22],[23]] and complaint duration on both clinical and biomechanical outcomes. In an attempt to minimise these, we aimed to recruit 36 participants to each group, and match them by age and gender, with an age boundary of 18–70 years and minimum complaint duration of 2 weeks. Anticipating a 20% loss to follow-up, this would provide 30 in each group. A sample size of 30 participants in each group would allow adequate opportunity for normal distributions of interval data if present [[24]] with a realistic recruitment target given the time and resources available. It would also give 80% power to detect a 3.5° (SD 6.5) increase in range in patients (which is the highest threshold for hypomobility, based on a review of plain film studies using the lower 2.5 percentiles of rotational range [[25]]) at the 95% level of significance.

#### Participants

Thirty patients who attended the Anglo-European College of Chiropractic’s (AECC) outpatient clinic for treatment of neck pain and who were suitable to receive a four-week (maximum eight sessions) course of spinal manipulation were recruited. Thirty pain-free healthy controls were recruited from staff, students and visitors to AECC and from the School of Health and Social Care a Bournemouth University. These were matched to patients for age and gender. The inclusion and exclusion criteria for patients and controls are shown in Table 1.

**Table 1:** Inclusion and exclusion criteria

All participants	Inclusion criteria: Male and female, Age 18 – 70 years, Able and willing to participate, No large radiological investigations or treatments in the past two years (effective dose > 10 mSv), Capable of giving informed consent, Not pregnant or likely to be pregnant, Willing for general practitioner to be informed about participation.
	Exclusion criteria: History of cervical spine surgery, Poor understanding of English, Current involvement as a subject in another research study.
Patients	Inclusion criteria: Mechanical neck pain (reproducible by neck movement/provocation tests) and no identifiable aetiology e.g. infection, inflammatory disease, Pain located within the area defined by the Neck Pain Task Force, Self-reported pain rating 3 or more on a 11-point numerical rating scale, Pain of at least 2 weeks duration, No contraindications to spinal manipulative therapy.
	Exclusion criteria: Non-mechanical neck pain, Depression history, Litigation/compensation pending, Manual therapy already received for this episode of neck pain, Primary complaint of arm pain, Traumatic onset of this neck pain episode, Central hypersensitivity as assessed by pressure algometry.
Healthy volunteers	Inclusion criteria: No activity-limiting neck pain lasting more than 24 hours in the last 12 months, No current neck pain, dizziness or vertigo (unsteadiness).
	Exclusion criteria: Cervical/thoracic spine manipulation in week prior to baseline imaging.

### Recruitment

Patients were identified by JB from first appointments at the AECC clinic for a new complaint of neck pain and were visited during their first attendance for assessment. If interested in participating, both patients and controls were assessed for eligibility using a pre-study form and were given information sheets that explained the study. If eligible and suitable for inclusion, all participants had at least 24 hours to reaffirm their decision to participate. Written informed consent was obtained from all participants and patients had all their treatments paid for from a study fund.

### Clinical assessment

Patients underwent a standard case history and examination by a final year chiropractic student intern. A further confirmatory examination was carried out by a chiropractic clinical tutor at which confirmation of suitability for a course of neck manipulation was sought and palpation findings for inter-vertebral motion were recorded. Informed consent, followed by the start of main data collection began prior to treatment, which commenced at the second visit. This included assessment for a history of diagnosed depression in the previous year and pressure algometry (Somedic Ltd, Sweden) for the assessment for pain central sensitisation. Ethical approval was granted by the UK National Research Ethics Service South West – Cornwall and Plymouth (11/SW/0072).

### Data collection

**Figure 1:**
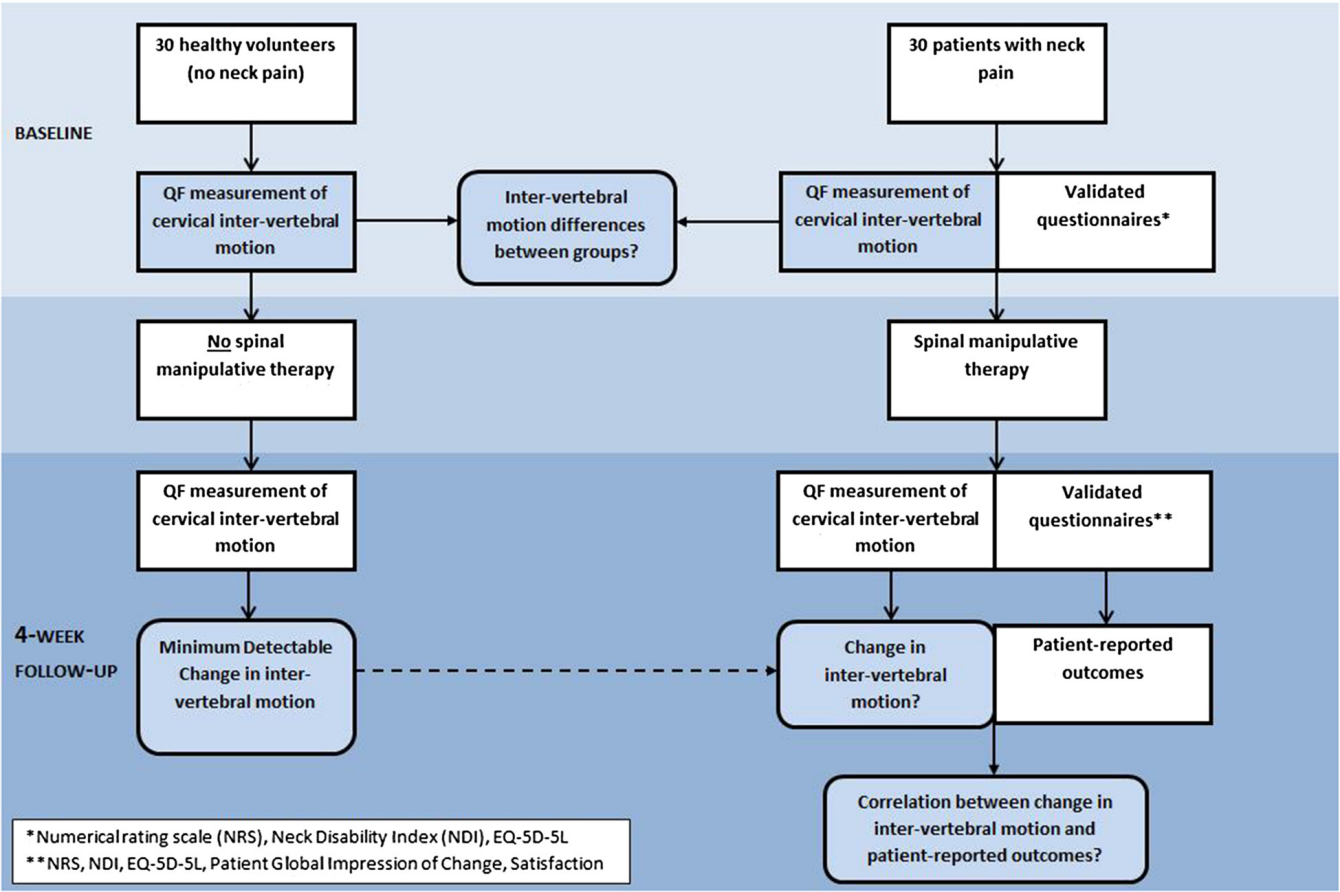
Study flow diagram.

The study proceeded according to the flow diagram in Figure 1. Before any treatment commenced, participants received QF screenings of cervical flexion and extension motion (separately) under a standardised protocol (described below). All screenings were conducted in the mid-mornings to avoid diurnal effects.

Patients completed an 11-point numerical rating scale for pain (NRS), the Neck Disability Index (NDI) [[26]], and the EuroQol EQ-5 L [[27]]. These, plus the Patient Global Impression of Change (PGiC) [[28],[29]] were completed again at 4 weeks along with a single satisfaction question [[30]]. The number of treatment visits, specific levels manipulated and their frequency were recorded for each patient. At 4 weeks, a second set of identical QF screenings were performed for all participants. Adverse events were recorded.

### Image sequence recording and analysis

**Figure 2:**
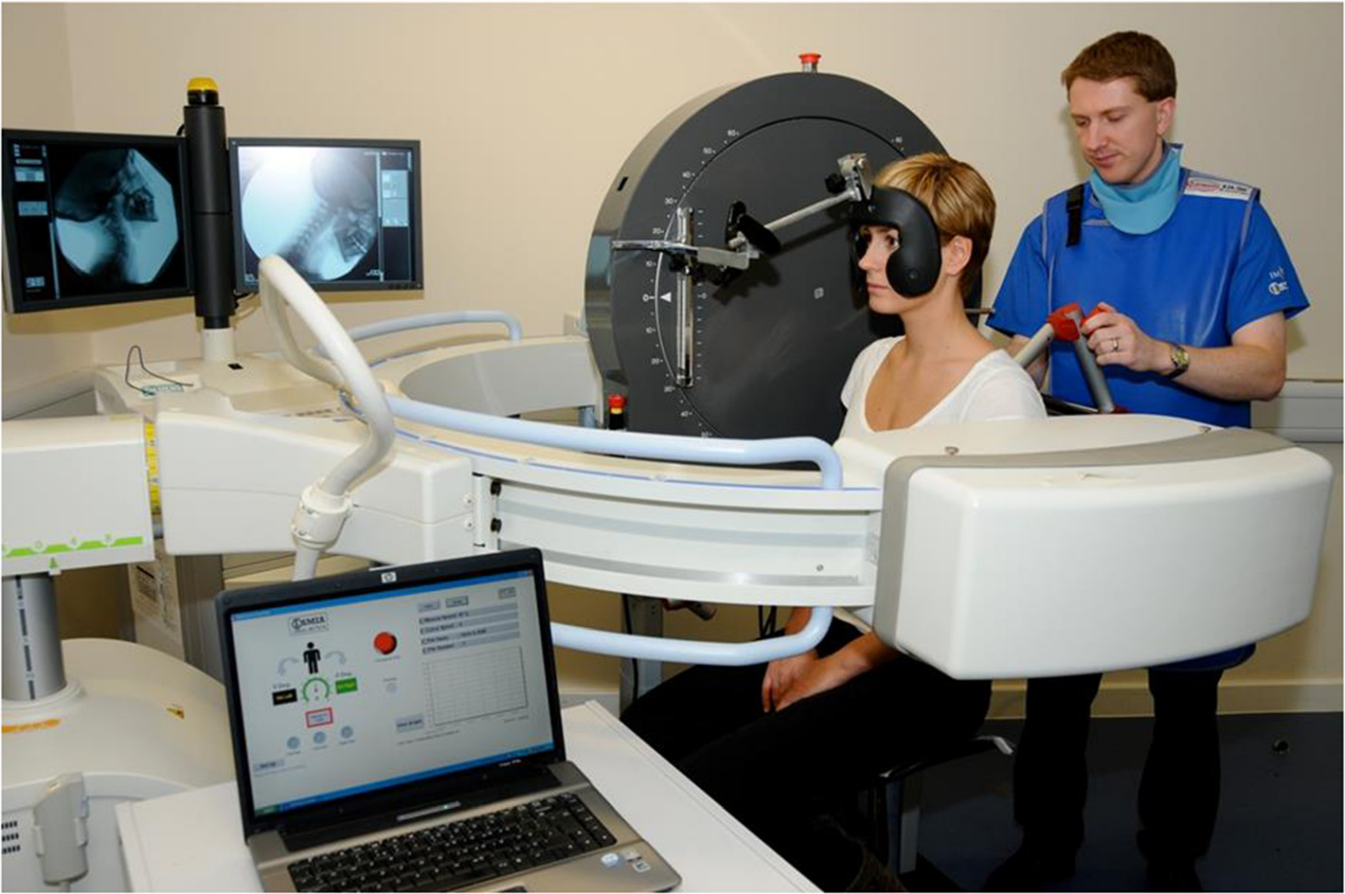
Fluoroscope and motion frame configuration.

The QF equipment consisted of a Siemens Arcadis Avantic VC10A digital fluoroscope (CE 0123, Siemens, Germany) and a vertical motion frame (Atlas Clinical Ltd. UK) with remote controller (Daqfactory Ltd. UK), stool and protective lead gonad shielding (Figure 2). After demonstration of the procedure, patients sat with their right shoulder next to the motion frame in a position that was judged ‘neutral’ to the operator using the infraorbitalmeatal line as a reference. This position was then fixed with sternal and thoracic (T5) bracing rods.

Participants completed a warm-up of 5 flexion-extension repetitions to reduce range variability [[31]] followed by recording of their maximum comfortable flexion and extension ranges using the CROM instrument (Performance Attainment Associates Inc. USA). A face rest rigidly attached by an extendable rod to the rotating disc section of the motion frame was brought to touch the patient’s cheeks with instructions to follow it during fluoroscopy, which was carried out with the central ray located in the C3-4 disc. The motion frame controller was set to reproduce the participants’ maximum attainable flexion and extension ranges after warm-up. This was synchronised with the QF screening procedure which recorded fluoroscopy sequences at 15frames per second during the motion. Recordings of measurements of the equipment positioning were made for duplication at follow-up. Radiation exposure data were recorded with a dose area product meter and effective dosage calculated using Monte Carlo simulation software (PCXMC) using the latest tissue weighting factors [[32]].

**Figure 3:**
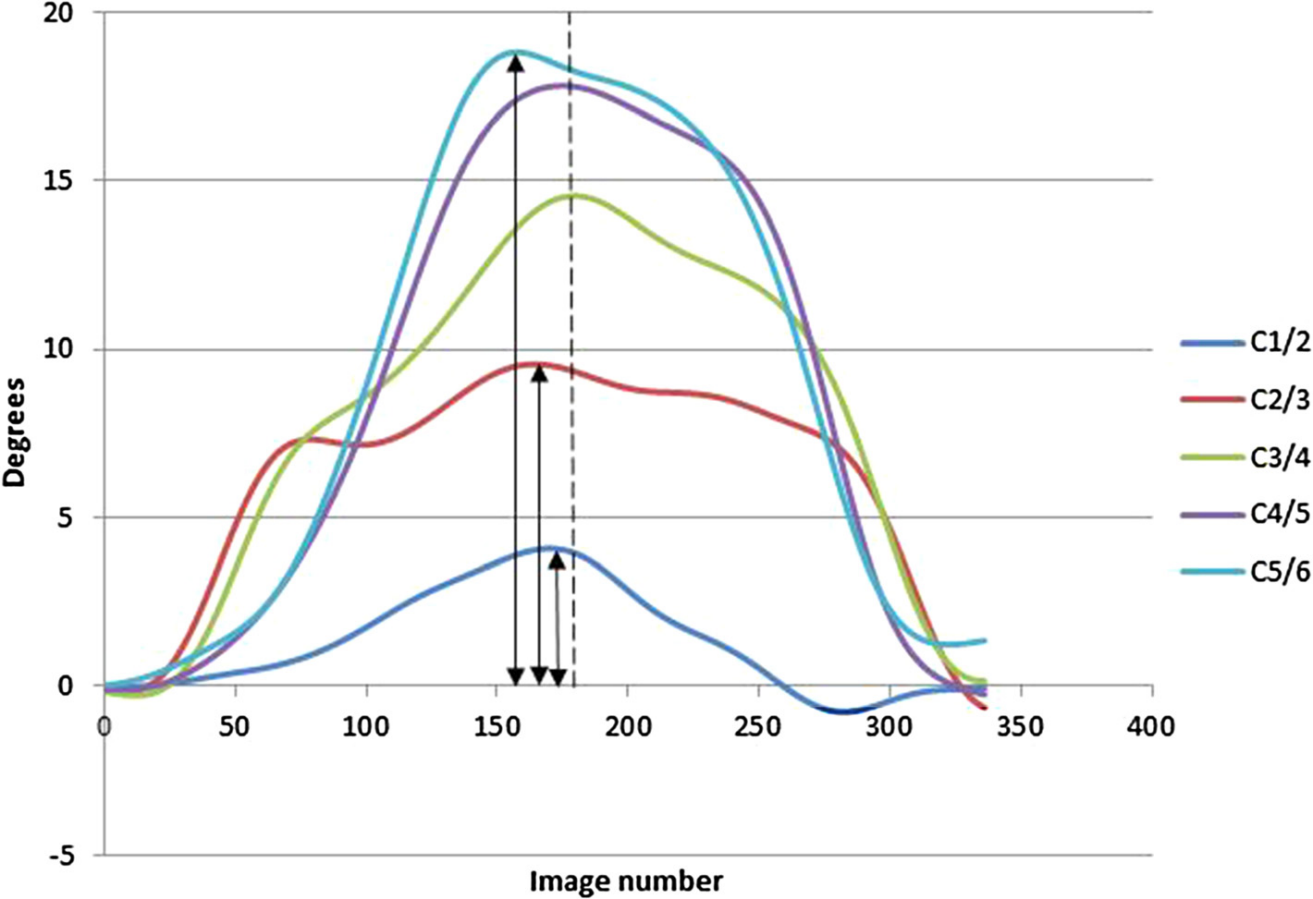
**Examples of IV-RoM compared to global motion.** IV-RoMs at individual levels are shown as peaks of coloured lines and the end of global motion as a dotted line.

Following screening, the image files (DICOM) were exported for analysis using bespoke image tracking and analysis codes written in Matlab (The Mathworks Ltd. UK) and accessed through a graphic user interface. The operator drew lines around the cortical margins of each vertebra from C1-C6 with the screen cursor to form tracking templates. These were linked to reference templates derived from 4-point vertebral body corner co-ordinates. This generated positional information for each vertebra and automatically calculated inter-vertebral rotations using a method similar to that used in the lumbar spine [[18]]. The output gave maximum IV-RoM data for each level and direction regardless of whether this occurred at the end of global range or not (Figure 3).

### Accuracy and repeatability of IV-RoM measurement

The accuracy of IV-RoM measurement using QF from continuous motion was determined using a model made from dry human C4-5 vertebrae joined at the vertebral body centres by a unidirectional plastic joint. This was mounted on a testing platform on the vertical motion frame and a digital inclinometer (Penny & Giles STT280; resolution +/−0.07°) was mounted on the superior vertebra to record tilting motion. The model was rotated by attaching its movable superior vertebra to a beam which was in turn rigidly attached to the rotating disc on the motion frame. This moved separately through 20° of flexion and 20° of extension at the same velocity as used for patient examinations. A block of minced beef was interposed between the X-ray source and the model to simulate soft tissue degradation. This test was repeated with the model rotated axially through 10° to invoke poor positioning. Maximum IV-RoM was measured 10 times by one observer for each range and orientation. Results were expressed as root-mean-square (RMS) differences between reference and index measurements.

Intra- and inter-observer repeatability was determined for inter-vertebral levels C1-6 in the first 10 participants recruited. Two trained observers (JB and a chiropractic student) independently analysed all 10 participants for maximum IV-RoM using the standard operating procedure for image analysis in the inter-observer study. JB then repeated the analysis of the same sequences 6 weeks later to determine intra-observer variation. Agreement was calculated for both tests as standard errors of measurement (SEM) [[33]] and intraclass correlation coefficients (ICC 2C,1 and 3C,1) for reliability [[34]].

### Interventions

Permitted treatments were given a maximum of twice a week for 4 weeks. These consisted of high velocity, low amplitude (HVLA) manipulation of the cervical spine using diversified techniques [[15]]. Myofascial trigger point therapy and/or light massage, hot/cold packs and analgesics were allowed if required. These are considered typical approaches for neck pain [[35]]. Mobilisation, passive or active muscle stretching and rehabilitation exercises were not allowed. Manipulation was carried out by qualified chiropractors with at least 5 years of practice experience.

### Data analysis

Age, gender and prevalence of radiographic anomalies were determined for both cohorts from baseline information, along with IV-RoM. At baseline, the latter was determined for all inter-vertebral levels in both directions from C1-6 in patients and controls and compared. Thresholds for designating motion at each level as ‘hypomobile’ were generated from four well-documented plain X-ray studies [[25],[36]–[39]], and summarised by Dietz et al. [[25]] Table 2.

**Table 2:** Accuracy, repeatability and thresholds for hypomobility (HT) and minimal detectable change (MDC) in IV-RoM in healthy volunteers

	**Flexion**		**Extension**	
**AccuracyRMS error (**^**o**^**) repeatability**	**In plane 0.21 intra-observer**	**Out of plane 0.50 inter-observer**	**In plane 0.34 intra-observer**	**Out of plane 0.40 inter-observer**
Agreement (SEM)				
C1-2	0.8	0.8	1.1	0.4
C2-3	0.3	0.4	0.8	0.7
C3-4	0.5	0.3	1.0	1.0
C4-5	0.6	0.5	0.8	0.8
C5-6	0.5	0.3	1.1	1.0
Reliability (ICC, 95% CI)	*	μ	*	μ
C1-2	0.97 (0.89-0.99)	0.96 (0.82-0.99)	0.90 (0.64-0.97)	0.97 (0.88-0.99)
C2-3	0.97 (0.90-0.99)	0.97 (0.90-0.99)	0.95 (0.80-0.99)	0.95 (0.71-0.98)
C3-4	0.99 (0.98-0.99)	0.99 (0.98-0.99)	0.92 (0.71-0.98)	0.97 (0.87-0.99)
C4-5	0.97 (0.89-0.99)	0.97 (0.89-0.99)	0.97 (0.89-0.99)	0.97 (0.85-0.99)
C5-6	0.99 (0.97-0.99)	0.99 (0.97-0.99)	0.97 (0.85-0.99)	0.97 (0.85-0.98)
Thresholds (^o^)	HT	MDC	HT	MDC
C1-2	1.3	3.8	1.1	4.4
C2-3	0.8	3.0	0.9	4.5
C3-4	1.8	3.9	2.3	6.4
C4-5	2.3	3.4	3.5	4.7
C5-6	1.1	3.4	1.3	5.0

* = ICC(3C,1), μ = ICC (2C,1), two-way single measure mixed effects model (consistency).

The minimal detectable change (MDC) in IV-RoM that could be expected due to measurement error in the absence of any intervention was calculated from control data as 2.77xSD of its intra-subject difference over 4 weeks [[40]]. IV-RoM changes beyond MDC for each level and direction were then calculated in patients. Correlations between the number of manipulations received by patients and the number of levels from C1-6 that increased beyond MDC were performed. Finally, correlations between IV-RoM changes beyond MDC in patients and NRS, NDI, EQ-5D changes, PGIC and satisfaction scores were determined.

All interval data were inspected for normality and statistical analyses were performed accordingly using SPSS (V21, IBM Software Ltd.) Differences in patients and controls were determined by unpaired t-tests, and between baseline and follow-up in patients by Wilcoxon Signed-Rank tests. Correlations between change scores and range increases were analysed using the Spearman correlation coefficient. Analysis of proportions of dichotomous variables was by 2-way Fisher Exact tests for comparison of cohorts and by Cohen’s Kappa statistic [[41]] for agreement between palpated and measured hypo-mobility. The cut-off for statistical significance was set at 0.05.

## Results

Of 191 patients approached, 30 were eligible and agreed to participate. Of these, 30 patients and 30 controls provided full documentation and imaging data. One patient’s imaging analysis failed and could not be recovered, therefore 29 patients and 30 healthy controls were analysed. Patients received a mean of 1.3 neck manipulations per visit (SD 0.4) and 10.7 over the course of the study (SD 3.5). The number of patients who received additional treatments was: trigger point therapy (27), light massage (27), hot or cold pack (7) and over the counter medication (18). Average radiation dose was (0.013 mSv), representing an additional cancer risk of 1:1 million [[42]].

### Accuracy and repeatability for range of motion, MDC and hypo-mobility cut-offs

Accuracy was high for IV-RoM (worst RMS error 0.5°) as was intra and inter-observer repeatability (highest SEM 1.1°, lowest ICC 0.90), Hypo-mobility cut offs ranged from 0.8° to 3.5° (Table 2).

The MDCs for flexion and extension IV-RoM over 4 weeks ranged from 3.0° (flexion at C2-3) to 6.4° (extension at C3-4). The MDC over the 4 week intervention period represents the smallest change that could not be attributed to normal variation and/or measurement error for these levels.

### Subject characteristics

The baseline characteristics of the participants are shown in Table 3. The average age of participants was 40 years (SD 13.1). Seventy percent were female, with no significant differences between patients and controls. In patients, the median duration of symptoms was 12 months (interquartile range 2–36) and 17/29 had pain at other sites. The mean baseline severity for pain and disability were NRS 5/10 and NDI 13/50 respectively. None had pain pressure thresholds below the reference norms [[43]].

**Table 3:** Baseline characteristics of participants

	**Patients**	**Healthy volunteers**	**Significance (p)**
N	29	30	
Age (years)	39.7 (13.1)	40.9 (13.1)	0.72†
Females	21/29	21/30	0.23‡
With skeletal variants/anomalies	9/29	5/29	0.23‡
Regional range of motion in flexion (degrees-SD)	49 (6.7)	53 (7.2)	0.04†
Regional range of motion in extension (degrees-SD)	51 (7.2)	56 (6.6)	0.03†
Median pain duration, weeks (interquartile range)	12 (2–36)		
With other pain sites	18/29	-	-
Mean pain pressure threshold, kPa (SD)	475 (160)	-	--
Mean pain score/10 (SD)	5 (1.5)	-	-
Mean NDI score/50 (SD)	13 (6.7)	-	-
Mean EQ-5D-5 L VAS/100	75 (15.5)	-	-
EQ-5D-5 L Index (SD)	0.74 (0.099)	-	-
Mean number of treatment visits (range)	8 (7–8)	-	-

*kPa*, kilopascals; *NRS*, Numerical Rating Scale for pain; *NDI*, Neck Disability Index; *EQ-5D-5 L*, Euroquol; *VAS*, Visual Analogue Scale; *†*, (unpaired) t test; *‡*, Fisher’s exact test.

#### Hypo-mobility

**Figure 4:**
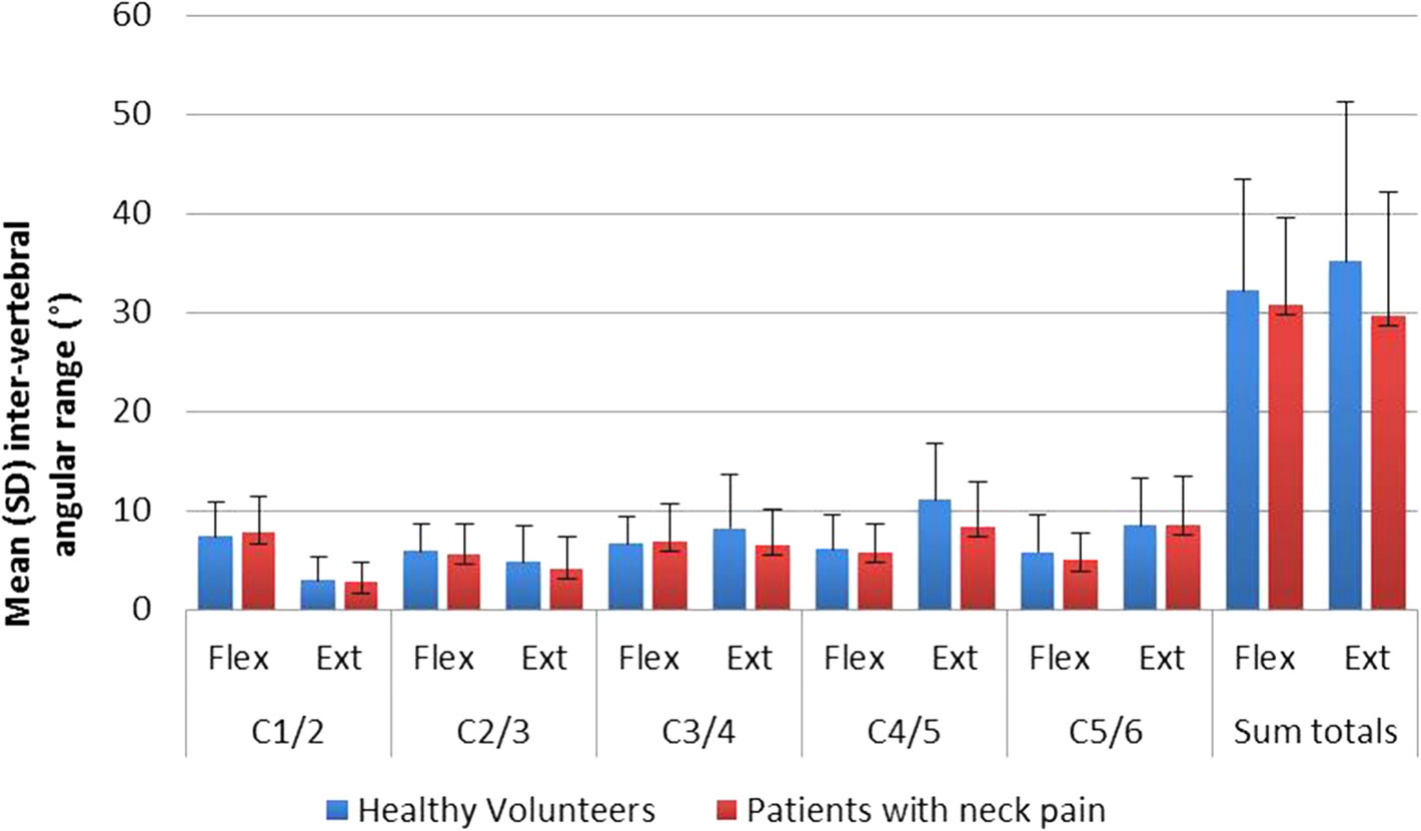
Baseline IV-RoM in patients and controls.

The baseline IV-RoMs in patients and controls are shown in Figure 4. Patients had on average less inter-vertebral motion in extension at levels between C1-6 (mean 5.7°) and less in flexion (mean 1.5°) at baseline than controls, but none were statistically significant (p > 0.05). There was also no significant difference between the number of hypo-mobile levels detected at baseline and follow-up in patients. Hypo-mobility was detected on measurement less frequently than on palpation and there was little agreement between examiner recognition of hypo-mobile segments and the frequency of being measured below hypo-mobiity thresholds, even when adjacent levels were included (Table 4).

**Table 4:** Hypomobile levels identified by palpation (C2-C5) and confirmed by measurement (n = 87)*

**Level**	**Identified hypomobile**	**Confirmed hypomobile**	**Percentage confirmed**	**Kappa (95% CI0**	**Significance (p)**
C2-3	14	6	43	0.00	0.500
C3-4	7	2	29	0.06 (−0.064-0.193)	0.163
C4-5	7	6	86	0.04 (−0.160-0.248)	0.336
Pooled	28	14	50	0.06 (−0.032-0.158)	0.096

*Number of hypomobile levels identified on palpation and confirmed by measurement as movement of <5 degrees in flexion or extension at the identified or adjacent level.

### Patient outcomes

A summary of patient outcomes is given in Table 5. Eighty-seven percent of patients had at least a 30% improvement, as reflected in PGiC scores, at the end of the treatment period. However, there was no relationship between any of the patient-reported outcomes and changes in IV-RoM beyond MDC at any level (Table 5). No serious adverse events occurred. However, temporary increases in symptoms were recorded at some point in the treatment period in 19/29 patients and headaches following treatment were recorded in 2/29. Most of these resolved within 24 hours and all were resolved by 96 hours. Two patients received treatments outwith the study protocol, consisting of manipulation to the thoracic spine at two treatment visits. One patient received cervical spine traction and muscle stretching techniques at one treatment visit.

**Table 5:** Correlations between change scores and levels that increased range above MDC in flexion or extension

**Measure/range**	**Baseline (SD)**	**Follow-up (SD)**	**Sig (p) †**	**% change (95% CI)**	Correlation % change and increased IV-RoM*	**Sig (p)**^**‡**^
NRS/10	5 (1.5)	2 (1.6)	p < 0.0001	52%	0.02 (−0.350- 0.383)	0.92
				(40.6-63.4)		
NDI/50	13 (6.7)	6 (4.9)	p < 0.0001	48%	0.12 (−0.260- 0.464)	0.54
				(36.2- 59.8)		
EQ-5D VAS/100	75 (15.5)	84 (14.9)	p = 0.001	6%	−0.12 (−0.465- 0.259)	0.54
				(−10.0- 22.0)		
EQ-5D Index	0.74 (0.10)	0.82 (0.11)	p < 0.0001	9%	−0.19 (−0.518- 0.192)	0.33
				(4.4- 13.6)		
PGIC/-10 to +10	-	-	-	87% improved**	−0.05 (−0.407- 0.325)	0.81

^*†*^Wilcoxon signed rank test; *‡*, Spearman’s rank correlation coefficient.

*Increased IV-RoM = number of inter-vertebral levels increased in range above MDC **At least 30% improvement.

#### Dose response

**Figure 5:**
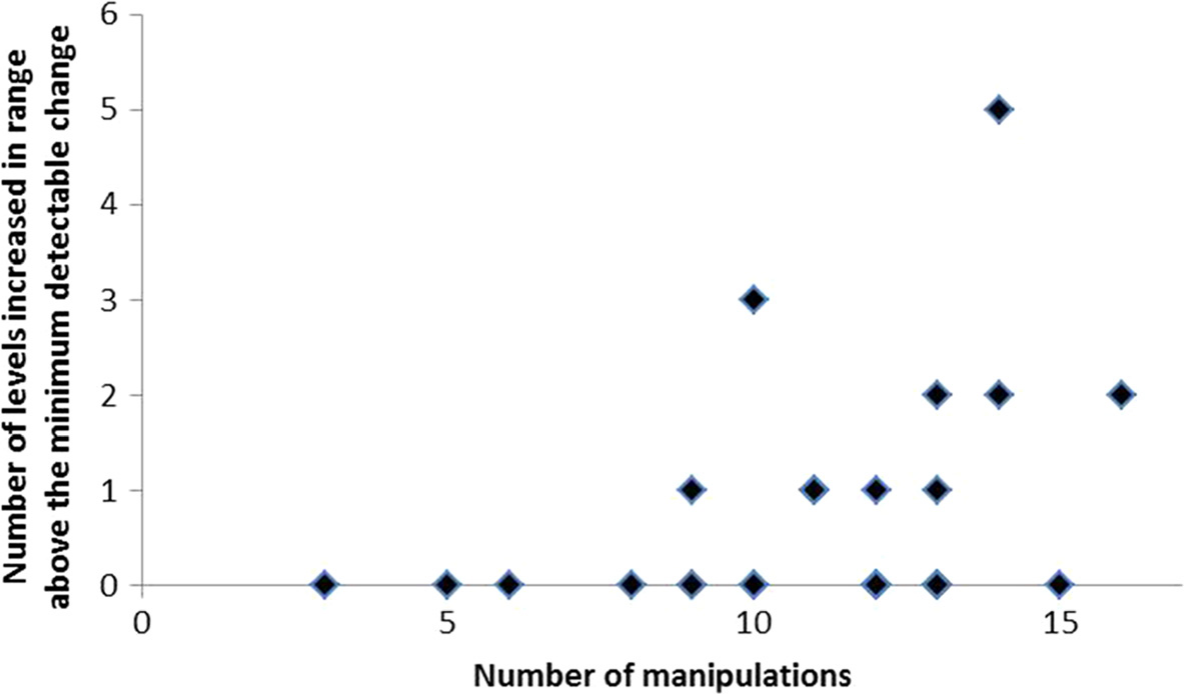
**Manipulations received by levels that increased in range.** Scatterplot showing the number of manipulations received against the number of intervertebral levels that increased their range above MDC. * = Spearman rank correlation.

There was a modest, but significant positive correlation between the number of manipulations received and the number of levels that increased in range at 4 weeks beyond their MDCs (Rho = 0.39, p = 0.043) (Figure 5). Furthermore, a significantly higher proportion of levels increased their IV-RoM in patients (13/16) who had at least 4 manipulations than at the same levels in controls (2/23, 2-sided p = 0.002 – Fisher exact). Most of these levels were not, however hypo-mobile at baseline. Of the patient levels that were hypo-mobile at baseline, only one increased its range by more than the MDC after treatment.

## Discussion

### Main result

Of importance is the finding that sagittal plane hypomobile segments were not significantly more prevalent in patients than in controls, casting doubt on the relevance of sagittal plane hypomobility in patients with relatively mild non-specific neck pain. However, this may not be the case in other populations and planes of motion. Indeed, such hypomobility may be of importance only in a subgroup of neck patients.

The lack of a relationship between symptomatic improvement and increased IV-RoM is also of interest. Clearly other mechanisms that improved the comfort and functional capacity of the patients in this study were in play, including spontaneous recovery. Other important biological factors may have included chemical factors in joint and muscle and activation patterns in the latter. However, this study seemed to rule out central pain hypersensitivity as a factor, as this was not detected at baseline in any of the patients. Psychological and social factors and their influence on functional behavior may also have had a role and may have been influenced by the interventions received.

Finally, it is of interest that there was a significant correlation (Rho = 0.39), albeit modest, between the number of manipulations received and the number of inter-vertebral levels that increased their IV-RoMs beyond MDC. Given the magnitude of some MDCs, it seems unlikely that this was a chance finding.

### Context of findings

There has been a growing body of research into motion-related dysfunction of the cervical spine. Many investigators have concluded that abnormal cervical spine kinematics, measured non-invasively, provides important diagnostic information in the evaluation of patients with neck disorders. Reduced range of regional motion, slow movement, repositioning errors, reduced coordination of movement, and lower peak velocities have all been demonstrated in chronic neck pain patients compared to controls [[44]–[47]]. Vogt et al. found that maximal cervical ROM was significantly lower, and movement variability significantly higher in chronic neck pain patients compared to healthy age-matched controls [[48]].

While some of these studies have identified what appear to be important motion differences in patients with spinal pain, the measurement methods used reflect regional motion, and do not reveal what is actually happening inside the neck [[49]] - or provide evidence about the extent to which differences are biomechanical or behavioural. The present study found little difference between patients and controls in terms of IV-RoM at baseline when participants were asked to reach their maximum range. Nevertheless, as with previous studies, patients had significantly lower voluntary regional motion ranges than controls at baseline (Table 3), which suggests that a behavioural element may be in play. However, it is difficult to imagine how the apparent dose–response effect at inter-vertebral levels in patients following manipulation could be anything other than mechanical when regional motion was set to the same range at follow-up as at baseline. This research therefore provides the first evidence suggestive of mechanical effects of treatment by manipulation at the inter-vertebral level. However, these changes were not related to patient reported outcomes.

### Limitations

QF was found to have high accuracy and repeatability for measuring sagittal plane IV-RoM in the cervical spine. However, high intra-subject variation across time at some levels (e.g. C3-4 in extension) will limit the ability of the present method, however accurate and precise, to measure changes in sagittal IV-ROM at some levels. Further refinement of the motion control protocol during image acquisition is therefore needed.

Our analysis only reports IV-ROM, and only in the sagittal plane. However, other kinematic parameters, such as translation, instantaneous axis of rotation and attainment rate (laxity) are also measurable with this method and some may be more responsive to change. Other planes of motion are also of interest, but are problematical for X-ray imaging due, for example, to the superimposition of the mandible and tracheal air space in the anterior-posterior projection and the lack of accessibility of axial or coupled motion with these methods. Palpation of the cervical spine for hypomobility is also typically carried out in all these directions, plus the oblique plane, thus providing a possible explanation for their more frequent detection by palpation and indicating the need for a 3-D method if this study is to be improved upon.

## Conclusion

This study found no differences in cervical sagittal IV-RoM between patients with mild non-specific neck pain and matched healthy controls and motion palpation over-estimated the prevalence of hypomobile segments. There was, however, a modest positive relationship between the number of spinal manipulations given and detectable increases in sagittal segmental IV-RoM. The patient-reported outcomes of spinal manipulation were not related to this, nor was there any relationship between increase in range and improvement in pain and disability, even though these improvements were generally large.

## Competing interests

The authors declare they have no competing interests.

## Authors’ contributions

This article is based on the PhD Thesis of JB, based on a concept generated by AB. All data were collected by JB assisted in part by AB. JB conducted all the analysis. Both authors contributed to the paper. Both authors read and approved the final manuscript.
